# Identifying pre-hospital factors associated with outcome for major trauma patients in a regional trauma network: an exploratory study

**DOI:** 10.1186/s13049-017-0419-4

**Published:** 2017-08-23

**Authors:** Lee Thompson, Michael Hill, Caroline Davies, Gary Shaw, Matthew D Kiernan

**Affiliations:** 1 North East Ambulance Service NHS Foundation Trust, Trauma Desk, Bernicia House, Goldcrest Way, Newcastle Upon Tyne, NE15 8NY UK; 2 Northumbria University, Coach Lane Campus, Newcastle Upon Tyne, NE7 7XA UK

**Keywords:** Pre-hospital, Trauma, Outcome, Age, Timings, Response

## Abstract

**Background:**

Major trauma is often life threatening and the leading cause of death in the United Kingdom (UK) for adults aged less than 45 years old. This study aimed to identify pre-hospital factors associated with patient outcomes for major trauma within one Regional Trauma Network.

**Method:**

Secondary analysis of pre-hospital audit data and patient outcome data from the Trauma Audit Research Network (TARN) was undertaken. The primary outcome used in analysis was ‘Status at Discharge’ (alive/deceased). Independent variables considered included ‘Casualty Characteristics’ such as mechanism of injury (MOI), age, and physiological measurements, as well as ‘Response Characteristics’ such as response timings and skill mix. Binary Logistic Regression analysis using the ‘forward stepwise’ method was undertaken for physiological measures taken at the scene.

**Results:**

The study analysed 1033 major trauma records (mean age of 38.5 years, SD 21.5, 95% CI 37–40). Adults comprised 82.6% of the sample (*n* = 853), whilst 12.9% of the sample were children (*n* = 133). Men comprised 68.5% of the sample (*n* = 708) in comparison to 28.8% women (*n* = 298).

Glasgow Coma Score (GCS) (*p* < 0.000), Respiration Rate (*p* < 0.001) and Age (*p* < 0.000), were all significant when associated with the outcome ‘Status at Discharge’ (alive/deceased).

Isolated bivariate associations provided tentative support for response characteristics such as existing dispatching practices and the value of rapid crew arrival. However, these measurements appear to be of limited utility in predictive modelling of outcomes.

**Discussion:**

The complexity of physiological indices potentially complicate their predictive utility e.g. whilst a Systolic Blood Pressure (SBP) of < 90 mmHg serves as a trigger for bypass to a Major Trauma Centre, the utility of this observation is nullified in cases of Traumatic Brain Injury.

Analysis suggested that as people age, outcomes from major trauma significantly worsened. This finding is consistent with existing research highlighting the relationship between trauma in elderly patients and poorer outcomes.

**Conclusion:**

Findings lend further validity to GCS, Respiration Rate and Age as predictive triggers for transport to a Major Trauma Centre. Analysis of interactions between response times, skill mix and triage demand further exploration but tentatively support the *‘Golden Hour’* concept and suggest a potential *‘load and go and play on the way’* approach.

## Background

Major trauma is often life-threatening and is the leading cause of death in the UK for adults under 45 years [1].

In April 2012, after reports identifying the need for specialist trauma care, Regional Trauma Networks (RTN) were introduced across the UK which enabled ambulance services to bypass local emergency departments and transport severely injured patients direct to definitive care at specialist Major Trauma Centres [2, 3].

Following the introduction of the local RTN a regional pre-hospital trauma registry was created. This data was combined with outcome data from the national trauma registry maintained by the Trauma Audit Research Network (TARN) [4]. TARN is a national organisation that collects and processes data on moderately and severely injured patients in England and Wales. TARN data allows networks, major trauma centres, trauma units, ambulance services and individual clinicians to benchmark their trauma service with other providers across the country. The combination of TARN with the RTN pre-hospital database enabled the creation of a meaningful dataset and allowed for a more comprehensive exploration of factors relating to pre-hospital trauma care. A key consideration in this analysis was understanding the epidemiology of a trauma system whilst taking into account the unique geographical features and demography of the region. Understanding the local regional major trauma epidemiology through this preliminary and exploratory study, with the intention of providing a baseline from which to evaluate future performance, would potentially identify trends and ultimately improve patient outcomes.

The aim of this study was to explore the pre-hospital casualty and response factors associated with major trauma outcomes in a RTN.

## Methods

**Fig. 1:**
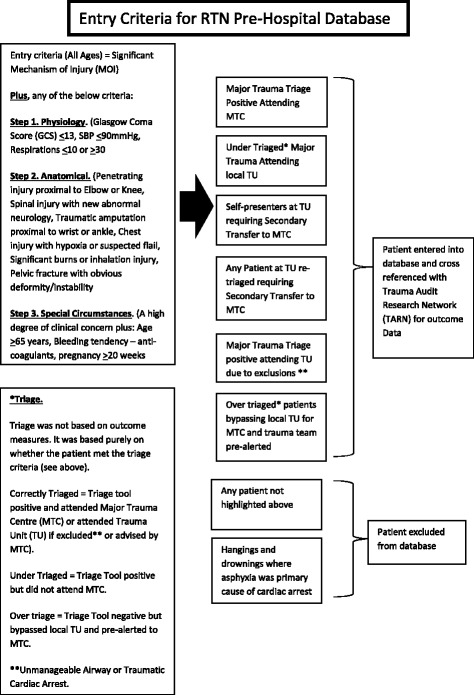
Criteria for patient entry into pre-hospital database

The study analysed combined data from TARN and RTN pre-hospital database for the North East (England) Ambulance Service producing a comprehensive dataset of regional major trauma patients. The entry criteria for patient inclusion within the RTN pre-hospital database can be seen in Fig. 1. Ethical approval for the study was granted via Northumbria University Research Ethics Review Panel. Reporting of the study followed the STROBE guidelines [5].

### Study period and population

The sample comprised of data collected between 1st of April 2012 and 30th September 2012 with each patient record within the dataset containing 69 variables. The study identified two groups of variables: ‘Casualty Characteristics’ obtained from patient care records, which included physiological measurements, age, MOI etc.; and ‘Response Characteristics’ obtained from Computer Aided Dispatch (CAD) records, which included response and transport times, crew skill mix and triage practices.

Within the sample there was a small number (*n* = 36, 3.5%) of casualties who were not classified as major trauma at initial triage, but were retrospectively included into the sample because they were later identified as meeting major trauma criteria. All ‘under-triaged’ patients managed at the Major Trauma Centres or Trauma Units were subsequently entered into the database.

The regional ambulance service covers an area of 8365 km^2^ serving over 2.71 million people in a mixed geography of rural and urban areas and receives over 1.5 million emergency and urgent calls per annum. The RTN, at the time of the study, had 9 Trauma Units and 2 Major Trauma Centres. There are 2 Helicopter Emergency Medical Service (HEMS) bases within the region which are charity-funded and each aircraft is staffed by Doctors qualified in Pre-Hospital Emergency Medicine (20 part-time doctors and 4 in training) and Paramedics (11 full-time equivalents). These aircraft do not fly at night, or when weather restricts visibility, but crews are able to respond to calls via a rapid response vehicle during these times. At the time of this study HEMS teams were available to respond on a Friday and Saturday night utilising a rapid response vehicle.

Paramedics within the RTN are trained and educated to carry out multiple interventions for trauma patients. These interventions include advanced airway management (endo-tracheal tube intubation and supraglottic airways), needle decompression of pneumothoraces and intravenous and intraosseous access. The application of haemorrhage control devices (tourniquets, haemostatic gauze) as well as Tranexamic Acid and immobilisation/splinting devices are also available to pre-hospital paramedics.

HEMS doctors within the region are also trained to manage cardiothoracic trauma up to and including resuscitative thoracotomy, peri-mortem C-section, rapid sequence induction (RSI) and the administration of blood products (although blood products were not on the aircraft at the time of this study).

Hazardous Access Response Team (HART) Paramedics are also available within the region to access patients at height, in water or in remote or difficult locations. At the time of the study there were 58 ambulance stations throughout the region with over 500 Paramedics who work alongside emergency care assistants and technicians.

### Data analysis

Descriptive statistics were used to characterise the study sample in terms of casualty and response characteristics. Categorical and ordinal variables were expressed as proportions and continuous variables expressed as means with standard deviations.

The primary outcome measure used in causal analysis was ‘Status at Discharge’ (alive/deceased). Independent variables were loosely grouped into two sets;

(i) casualty characteristics e.g. age and physiological indices, and

(ii) response characteristics e.g. skill mix and transport time.

**Fig. 2:**
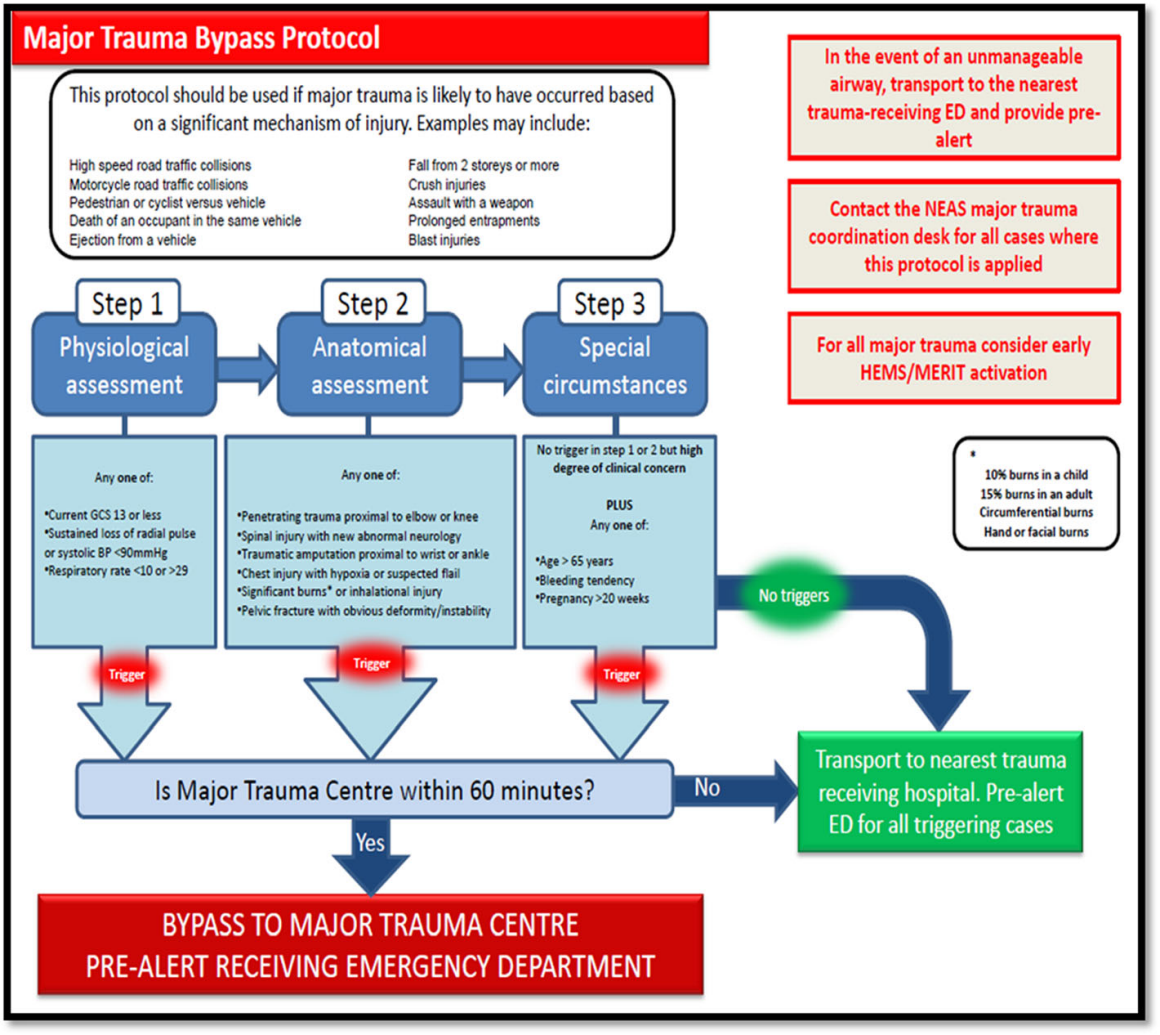
Major trauma triage protocol

Whilst there were multiple recordings of physiological indices for most patients within the study, the set employed for analysis purposes were the observations used by the attending crew in their pre-alert or alternatively, those observations that prompted the use of the major trauma triage tool shown in Fig. 2. Preliminary bivariate analysis was undertaken in order to explore relationships between these factors and outcome ‘Status at Discharge’ (alive/deceased).

To adjust for collinearity and potential amplification bias a binary logistic regression analysis was undertaken with the outcome ‘Status at Discharge’ (alive/deceased) as the dependant variable. Only independent variables that were individually associated with the outcome ‘Status at Discharge’ (alive/deceased), at a *p* ≤ 0.05 level were entered into the binary logistic regression model. All candidate predictor variables were entered into the model using a forward stepwise method, and each variable’s contribution to the overall fit was estimated using likelihood ratio tests. Analyses were undertaken using the Software Package for the Social Sciences (SPSS; Version 22, IBM Inc.; Armonk, NY, USA).

## Results

The study sample consisted of 1033 patient records that met the pre-hospital major trauma triage protocol criteria, as shown in Fig. 2. Table 1 highlights descriptive analysis of demographic characteristics of the sample group and key emergency response characteristics.

**Table 1:** Demographics, crew attendance, timings and triage

Age in years	Years [SD] (95% CI)
Mean	38.5 [21.5] (37–40)
Missing	86
Adult/Child <17	n (%)
Adult	853 (82.6)
Child <17	133 (12.9)
Missing	43 (4.2)
Sex	n (%)
Male	708 (68.5)
Female	298 (28.8)
Missing	23 (2.2)
Pre-hospital Traumatic Cardiac Arrest (TCA) – Active Resuscitation	n (%) 30
Died on scene	7 (23)
Transported to hospital	23 (77)
Alive at discharge	3 (10)
Died in hospital	18 (60)
Missing	2 (7)
In hospital deaths from pre-hospital major trauma (Not pre-hospital TCA)	n (%) 43 (4)
Attended by	n (%)
HEMS	168 (16.3)
Land Crews	844 (81.7)
Missing	17 (1.6)
Crew arrival times	Minutes [SD] (95% CI)
Mean	12 [13.5] (11–13)
HEMS	14 [11.5] (12–16)
Land Crews	11.5 [13.5] (10.5–13)
On scene times	Minutes [SD] (95% CI)
Mean	36 [19.5] (35–37.5)
HEMS	51.5 [21.5] (48–55.5)
Land Crews	33.5 [18] (32.5–35)
Transport time	Minutes [SD] (95% CI)
Mean	17 [11.5] (16–17.5)
HEMS	13 [9.5] (11–14.5)
Land Crews	17.5 [12] (16.5–18.5)
Time to definitive care (999 call to arrival at receiving facility)	Minutes [SD] (95% CI)
Mean	65 [27] (63–66.5)
HEMS	78 [23] (73.5–81.5)
Land Crews	62.5 [27] (60.5–64.5)
Triage	n (%)
Correct triage	744 (72.0)
Under triage	36 (3.5)
Over triage	248 (24)
Missing	5 (0.5)

Bivariate analysis revealed that a number of variables were significantly associated with outcome ‘Status at Discharge’ (alive/deceased) shown in Tables 2 and 3.

**Table 2:** Relationship of Independent variables associated with outcome ‘Status at discharge’ (Alive/Deceased), obtained using bi-variate analysis

Independent Variable	Test Statistic	*df.*	p	Mean Difference	95% CI(Lower)	95% CI(Upper)
GCS	*t* = −10.222	44.15	*p* ≤ 0.000	7.28 scale points	5.845	8.715
Respiratory Rate	*t* = −5.241	43.55	*p* ≤ 0.000	8 per min.	5	12
Systolic BP at Scene	*t* = −3.027	37.52	*p* ≤ 0.004	11.4 mmHg	11.46	57.7
Age	*t* = −5.464	738	*p* ≤ 0.000	17.8 years	11.47	24.24
Transport Time	*t* = −2.085	685	*p* ≤ 0.037	−3.78 min	−7.14	- 0.21
Skill Mix	u = 18,239	-	*p* ≤ 0.036	-	-	-
Triage	u = 19,959		*p* < 0.000	-	-	-

**Table 3:** Results from binary logistic regression analysis of predictor variables for outcome ‘Status at Discharge’ (alive/deceased)

	Significant Variables within the predictive model			
	Variable	Wald	P	Odds Ratio
Step 3	GCS	39.662	*p* ≤ 0.000	1.587 (95% CI: 1.374–1.833)
	Age	25.097	*p* ≤ 0.000	0.923 (95% CI: 0.894–0.952)
	Respiratory Rate	11.553	*p* ≤ 0.001	1.165 (95% CI: 1.067–1.272)

### Binary logistic regression analysis

In order to address problems of collinearity and the potential for amplification bias, binary logistic regression was undertaken for 571 casualties using outcome ‘Status at Discharge’ (alive/deceased) as the dependant variable. Analysis proceeded on the basis of ‘listwise’ exclusion, and this resulted in a total of 462 cases being excluded from the analysis sample (*N* = 1033). Variables were entered into the model on the basis of the ‘forward stepwise’ method. All candidate variables considered for inclusion in the binary logistic regression model were individually associated with the outcome ‘Status at Discharge’ (alive/deceased) at the *p* ≤ 0.050 significance level.

A test of the full model against a constant only model was statistically significant, indicating that the predictors as a set reliably distinguished between alive or deceased (χ2 = 103.862, *p* ≤ 0.000). Collectively, all seven candidate predictors “explained” 94% of the variability in ‘Status at Discharge’ (alive/deceased).

Step 1 Nagelkerke’s R^2^ (45.8%) the model included ‘GCS’ score only and indicated a moderate relationship between prediction and grouping.

Step 2 Nagelkerke’s R^2^ (59.8%) the model included ‘GCS’ score and ‘Age’ and indicated a stronger relationship between prediction and grouping.

Step 3 the final model included ‘GCS’, ‘respiratory rate’, and casualty’s ‘age’ and indicated that these factors are significant predictors of outcome ‘Status at Discharge’ (alive/deceased) (χ2 = 155.902, *p* < 0.000). The other four candidate predictors, ‘transport time’, ‘triage’, ‘skill mix’ and ‘systolic BP’, were not significant.

‘GCS’, ‘age’ and ‘respiratory rate’ were all significant at the 5% level (‘GCS’ – *p* < 0.000; ‘age’ - *p* < 0.000; ‘respiratory rate’ - *p* < 0.001).

The odds ratio (OR) were as follows: ‘GCS’ was 1.587 (95% CI: 1.374–1.833); ‘Age’ was 0.923 (95% CI: 0.894–0.952); ‘respiratory rate’ was 1.165 (95% CI: 1.067–1.272). The model correctly predicted 99.3% of the variability of an ‘Alive’ outcome, and 67.6% of ‘Deceased’ outcome at discharge, giving an overall percentage correct prediction rate of 97.4%.

## Discussion

The findings of this study suggest that physiological measures taken at the scene are of greater predictive utility than are emergency services response characteristics. Specifically, GCS, respiration rate, and age formed significant elements of the predictive model. Further analysis suggested that as people age, outcomes from major trauma significantly worsened. This finding is consistent with existing research highlighting the relationship between trauma in elderly patients and poorer outcomes [6–8].

Almost all previous studies with regard to physiology and trauma were exclusive to the in-hospital setting [9–12]. The evaluation of pre-hospital physiological variables as predictors of trauma outcome has, hitherto, been neglected. However, it should be noted that the inherent complexity of physiological indices (and their significance) potentially complicate their predictive utility e.g. whilst a Systolic Blood Pressure (SBP) of <90 mmHg serves as a trigger for bypass to a Major Trauma Centre, the utility of this observation is nullified in cases of Traumatic Brain Injury (TBI). Recent research [13] has identified that each 10 mmHg reduction in SBP is associated with an 18% increase in mortality when SBP falls below 120 mmHg. These findings should make us reconsider the hypotensive threshold for the isolated TBI patient group.

Emergency services response characteristics most often form the basis for Key Performance Indicators (KPI’s) for ambulance service delivery and evaluation: For example, the UK Department of Health requires ambulance services within England to respond to Red (life threatening calls) within 8 min regardless of rural or urban location [14]. The rise of evidence-based medicine has brought with it the unintentional consequence of ‘therapeutic nihilism’ [15], in which failure to establish supporting evidence for an intervention is (incorrectly) interpreted as a warrant for therapeutic inertia. The pressures to ‘do nothing’ are further exacerbated during times of fiscal austerity, where being unable to unequivocally establish an evidence base can underpin the further erosion of service standards such as crew response times. In terms of the current study, it would appear that (abnormal) physiological measurements, possibly as a reflection of the severity of trauma and nature of the physiological insult, underpin the most accurate predictive model of mortality outcomes.

Existing research considering the impact of timings and skill mix is markedly limited to consideration of on-scene times and predominately focused on the impact of physician led teams in prolonging ‘on-scene’ times [16–19]. These studies have typically added fuel to the ‘stay and play’ or ‘load and go’ debate and highlight the ‘golden hour’ of immediate care [20, 21]. However, the typical lack of standardisation in how ‘on-scene’ times are defined and recorded raises valid questions concerning the potential generalisability of these findings beyond the context in which individual studies were undertaken. In the context of the current study, HEMS teams took longer to arrive on scene, compared to land based resources, had extended ‘on-scene’ times and longer overall mean time from emergency call to arrival at receiving facility. As previously noted, HEMS teams have a wider scope of practice and are able to initiate additional interventions such as RSI. More widely, the huge variation in scope of practice between professions in different regions and countries [22] makes direct comparisons difficult. Although pre-hospital timings were extended for HEMS teams, compared to land based teams, this study did not examine whether the presence of HEMS improved timings for ongoing management such as time to theatre or time to Computerised Tomography (CT) scan [23].

Significantly, within this study it was noted that those major trauma casualties who were correctly triaged were more likely to have poorer outcomes. This finding may suggest that crews are using existing triage practices in order to correctly classify trauma severity. Whilst some patients are incorrectly over triaged and transported to a Major Trauma Centre and some major trauma patients inappropriately under triaged and transported to local hospital emergency departments, these eventualities did not appear to be statistically associated with significantly adverse outcomes. The tendency to over-refer casualties to Major Trauma Centres is perhaps an artefact of the precautionary principal in action.

## Limitations

The study was conducted during the spring and summer months and does not account for seasonal variations which may have affected mechanism of injury, available flying time (daylight in northern UK is 18 h in the summer and 6 h in winter) and driving conditions due to adverse weather during the winter months (snow and ice). There is a strong likelihood of ecological and confounding relationships within the current data set. Whilst bivariate analysis such as is reported above can reveal interesting associations, the large number of degrees of freedom involved, the potential for collinearity and amplification bias by means of multiple comparisons using the same variables would risk Type I errors.

As is the case with all uses of secondary data, analysis is constrained by the fact that data are collected for purposes other than the researcher’s intentions [24]. Furthermore, the large number of personnel involved in data collection inevitably potentiates the risk of poor inter-rater reliability [25]. Whilst mortality data serves as an absolute binary outcome measure, morbidity data is less tangible [26]. Further research is required in order to understand the utility of the predictor variables considered in this paper in predicting morbidity outcomes, especially in the case of life-changing morbidity.

## Conclusions

This study identifies that local pre-hospital major trauma predominantly affects the male population with the mean age of 38.5 years. Further research is required in order to more fully understand the predictive utility of age in determining major trauma outcomes and possibly to allow the generation of age-specific triage criteria.

Contrary to current English ambulance performance targets, this study identified that there is greater predictive utility in relation to outcome from physiological measures taken at the scene than emergency services response times which, paradoxically, comprise key performance indicators for service delivery.

Rather than interpreting this finding as a warrant for the therapeutic nihilist instruction to abandon targets, we conclude that further analysis is required in order to establish the value of response characteristics in relation to morbidity outcomes and the alleviation of suffering.

The authors tentatively suggest that given the lack of evidence for emergency services response characteristics as predictors of mortality outcomes, a ‘*load and go and play on the way*’ approach to patient transport might be advocated.

## Abbreviations

CAD: Computer aided dispatch
CT: Computerised tomography
GCS: Glasgow coma score
HART: Hazardous access response team
HEMS: Helicopter emergency medical service
KPI: Key performance indicator
MERIT: Medical emergency response incident team
MOI: Mechanism of injury
MTC: Major trauma centre
NEAS: North east ambulance service
NHS: National health service
RSI: Rapid sequence induction
RTN: Regional trauma network
SBP: Systolic blood pressure
TARN: Trauma audit research network
TBI: Traumatic brain injury
UK: United Kingdom

## References

[1] Sukumaran S, Henry JM, Beard D, Lawrenson R, Gordon MW, O'Donnell JJ, Gray AJ (2005). Prehospital trauma management: a national study of paramedic activities. Emerg Med J.

[2] National Confidential Enquiry into Patient Outcome and Death. Trauma: who cares? : a report of the National Confidential Enquiry into Patient Outcome and death (2007). London: National Confidential Enquiry into Patient Outcome and Death (NCEPOD); 2007.

[3] National Audit Office (2010). Major trauma care in England.

[4] Trauma Audit and Research Network: TARN. [https://www.tarn.ac.uk/]. Accessed 18 Aug 2017.

[5] Vandenbroucke JP, von Elm E, Altman DG, Gøtzsche PC, Mulrow CD, Pocock SJ, Poole C, Schlesselman JJ, Egger M, S.I (2007). Strengthening the reporting of observational studies in epidemiology (STROBE): explanation and elaboration. PLoS Med.

[6] Cox S, Morrison C, Cameron P, Smith K (2014). *Advancing age and trauma: Triage destination compliance and mortality in Victoria,* Australia. Injury.

[7] Kehoe A, Smith JE, Bouamra O, Edwards A, Yates D, Lecky F (2016). Older patients with traumatic brain injury present with a higher GCS score than younger patients for a given severity of injury. Emerg Med J.

[8] Kehoe A, Smith JE, Edwards A, Yates D, Lecky F (2015). The changing face of major trauma in the UK. Emerg Med J.

[9] Lin G, Becker A, Lynn M (2011). Changes in vital signs of trauma victims from prehospital to hospital settings. J Paramedic Pract.

[10] Victorino GP, Battistella FD, Wisner DH (2003). Does tachycardia correlate with hypotension after trauma?. J Am Coll Surg.

[11] Ocak G, Sturms LM, Hoogeveen JM, Le Cessie S, Jukema GN (2009). Prehospital identification of major trauma patients. Langenbeck's Arch Surg.

[12] MacLeod JBA, Maurico L, McKenney MG, Jeroukhimov I, Cohn SM (2004). Predictors of mortality in trauma patients. Am Surg.

[13] Spaite DW, Hu C, Bobrow BJ (2017). Mortality and prehospital blood pressure in patients with major traumatic brain injury: implications for the hypotension threshold. JAMA Surg.

[14] Turner J, O’Keeffe C, Dixon S, Warren K, Nicholl J (2006). The costs and benefits of changing ambulance service response time performance standards.

[15] Starr P (1976). The politics of therapeutic nihilism. The new critics of medical care. Hast Cent Rep.

[16] Carr BG, Caplan JM, Pryor JP, Branas CC (2006). A meta-analysis of prehospital care times for trauma. Prehosp Emerg Care.

[17] Carr BG, Brachet T, David G, Duseja R, Branas CC (2008). The time cost of prehospital intubation and intravenous access in trauma patients. Prehosp Emerg Care.

[18] Di Bartolomeo S, Valent F, Rosolen V, Sanson G, Nardi G, Cancellieri F, Barbone F (2007). Are pre-hospital time and emergency department disposition time useful process indicators for trauma care in Italy?. Injury.

[19] Dissmann PD, Le Clerc S (2007). The experience of Teesside helicopter emergency services: doctors do not prolong prehospital on-scene times. Emerg Med J.

[20] Harmsen AMK, Giannakopoulos GF, Moerbeek PR, Jansma EP, Bonjer HJ, Bloemers FW (2015). The influence of prehospital time on trauma patients outcome: a systematic review. Injury.

[21] Lerner EB, Moscati RM (2001). The golden hour: scientific fact or medical “urban legend”?. Acad Emerg Med.

[22] Eckstein M, Chan L, Schmeir A, Palmer R (2000). Effective prehospital advanced life support on outcomes of major trauma patients. Am J Emerg Med.

[23] Garner AA, Mann KP, Poynter E, Weatherall A, Dashey S, Puntis M, Gebski V (2015). Prehospital response model and time to CT scan in blunt trauma patients; an exploratory analysis of data from the head injury retrieval trial. Scand J Trauma Resusc Emerg Med.

[24] Bulmer M (1980). Why Don't sociologists make more use of official statistics?. Sociology.

[25] Cicchetti DV (1976). Assessing inter-rater reliability for rating scales: resolving some basic issues. Br J Psychiatry.

[26] Bowling A (2004). Measuring health: a review of quality of life measurement scales.

